# Dental Pathology and Surgery

**Published:** 1875-04

**Authors:** 


*Dental Pathology and Surgery.* By S. James A. Salter, M. B., F. R. S. New York: Wm. Wood & Co., 1875. Buffalo: H. H. Otis.

The author has gathered in this work many of his former essays, and has arranged them under suitable heads. The book may be considered, therefore, as the out-growth of the experience of twenty-three years practice in this special branch of surgery. The volume is composed of twenty-eight chapters, the first two of which are devoted to the general anatomy of the teeth and their various functions. The succeeding chapters treat of supernumary teeth, deficiencies, irregularities, secondary dentine, congenital defects, mechanical injuries, cavities, diseases of the teeth and associate parts, etc. The whole subject is admirably treated, and the work will prove valuable to general practitioners, as they are often called upon to express opinions in which a knowledge of the contents of this work would be of material benefit. The chapters upon the extraction of teeth are especially valuable in this respect, as it is an operation which physicians residing at a distance from a dentist are often called upon to perform. The author has most certainly succeeded in placing the results of his extended experience before the profession in a most servicable form.

